# Early Risk Stratification of Delirium in Intermediate Care Units and Its Impact on 30-Day Mortality: A Decision-Tree Analysis

**DOI:** 10.3390/clinpract16070131

**Published:** 2026-07-13

**Authors:** Fabrizio Lucente, Lucia Filippi, Arian Zaboli, Alessandra Eugenia Bionda, Michael Maggi, Marta Parodi, Alice Bresolin, Arianna Pretto, Martina Da Meda, Silvia Greselin, Francesca Fulghesu, Gianni Turcato

**Affiliations:** 1 Intermediate Care Unit, Department of Internal Medicine, Hospital Alto Vicentino (AULSS7), 36014 Santorso, Italy; fabrizio.lucente@aulss7.veneto.it (F.L.); michael.maggi@aulss7.veneto.it (M.M.); marta.parodi@aulss7.veneto.it (M.P.); alice.bresolin@aulss7.veneto.it (A.B.); arianna.pretto@aulss7.veneto.it (A.P.); martina.dameda@aulss7.veneto.it (M.D.M.); silvia.greselin@aulss7.veneto.it (S.G.); francesca.fulghesu@aulss7.veneto.it (F.F.); gianni.turcato@aulss7.veneto.it (G.T.); 2 Health Professions Management, South Tyrolean Health Authority (SABES-ASDAA), 39100 Bolzano, Italy; zaboliarian@gmail.com; 3 Department of Clinical and Experimental Medicine, University of Pisa, 56126 Pisa, Italy; a.e.bionda@gmail.com; 4 Department of Health Sciences, UniCamillus–Saint Camillus International University of Health Sciences, 00131 Rome, Italy

**Keywords:** delirium, IMCU, early risk factors, prognostic

## Abstract

Background: Delirium is an under-recognized complication in hospitalized acute care patients, associated with worse outcomes including increased length of stay, higher ICU admission rates and greater mortality. Although screening tools exist for early diagnosis, the absence of admission-based tools limits early risk stratification and timely prevention strategies. Aim: To identify early risk factors for delirium development in IMCU patients and quantify its prognostic role on 30-day mortality. Methods: In this prospective single-center study, 651 consecutive IMCU patients without delirium at admission were enrolled. Admission variables were analyzed using multivariable logistic regression and Classification and Regression Tree (CART) analysis to identify clinically relevant risk phenotypes. The association between delirium and 30-day mortality was assessed in adjusted models. Results: Delirium developed in 18.6% of patients within 96 h. Key independent risk factors included age (OR 1.04), male sex (OR 1.75), alcohol use disorder (OR 3.59), cognitive impairment (OR 3.35), COPD (OR 1.68), NEWS (OR 1.18), and need for NIV (OR 3.83). CART analysis identified NIV as the dominant early discriminator, followed by cognitive vulnerability and acute severity. 30-day mortality was significantly higher in patients with delirium (22.3% vs. 9.8%, *p* = 0.001). Delirium remained an independent risk factor after adjustment (OR 2.53). CART analysis further corroborates delirium as a significant determinant, enhancing prognostic stratification beyond its role as a mere surrogate of disease severity. Conclusions: Admission-level clinical variables and exploratory CART analysis identified clinically interpretable delirium risk phenotypes in IMCU patients. Delirium was independently associated with increased 30-day mortality. These findings provide a preliminary framework for future validation studies and development of IMCU-specific risk-stratification approaches.

## 1. Introduction

Delirium is an acute neuropsychiatric syndrome characterized by altered attention and awareness, rapid onset and a fluctuating course, associated with acute medical conditions [1,2]. It is a frequent complication in hospitalized patients, with a prevalence of 20–50% in acute medical wards and up to 80% in critically ill patients admitted to the intensive care unit (ICU) [3,4], and is associated with increased length of stay, healthcare costs, and worse clinical outcomes, including ICU transfer and in-hospital mortality [4,5].

Several tools, including the CAM and 4AT for general medical settings and the CAM-ICU or ICDSC for intensive care settings, have been developed for early delirium recognition across different care environments [6,7,8,9,10]. However, these tools are primarily diagnostic in nature and do not allow identification, at the time of admission, of patients at high risk of developing delirium in the subsequent hours or days. This limitation is particularly relevant because, once established, delirium is difficult to treat, whereas preventive interventions—both pharmacological and non-pharmacological—are more effective when initiated early and targeted at high-risk patients [4,11,12,13].

This gap is even more pronounced in the context of Intermediate Medical Care Units (IMCUs), which represent an intermediate level of care between general wards and ICUs and admit patients characterized by high vulnerability and clinical instability [14,15]. Despite the plausibly significant risk of delirium in this setting, data on its incidence, determinants, and prognostic impact remain limited and are largely extrapolated from ICU or general ward populations. In this context, the lack of risk-stratification tools applicable at admission may hinder early identification of high-risk patients and timely implementation of targeted preventive strategies. Therefore, the development of IMCU-specific risk-stratification approaches based on clinical variables available at admission may represent a valuable tool for early risk stratification and for guiding timely, targeted preventive interventions in this setting.

The primary aim of this study was to identify clinical characteristics available at IMCU admission associated with the risk of developing delirium. The secondary aim was to evaluate the association between incident delirium and clinical outcomes in IMCU patients.

## 2. Materials and Methods

### 2.1. Study Design and Setting

This was a prospective single-center observational study conducted in the Medica IMCU of Alto Vicentino Hospital, Santorso (Italy), between 1 January and 31 December 2024.

The IMCU is a high-dependency unit within the Department of Internal Medicine, dedicated to the management of acutely ill patients requiring close clinical monitoring and non-invasive organ support, without immediate indication for ICU admission or invasive organ replacement therapies.

The unit admits patients from the Emergency Department (ED) or general wards presenting with clinical instability not adequately manageable in standard care settings and requiring a higher level of care, including continuous multiparametric monitoring, advanced oxygen therapy, non-invasive ventilation, or vasoactive support. The primary goal of the IMCU is early stabilization of acute conditions, typically within the first 96 h after admission.

The study was approved by the local Ethics Committee and conducted in accordance with the Declaration of Helsinki. Written informed consent was obtained from all patients or their legal representatives.

### 2.2. Patients

All consecutive patients admitted to the IMCU during the study period were considered eligible.

Patients were excluded if they had an ongoing episode of acute delirium at IMCU admission, defined as a positive Confusion Assessment Method for the Intensive Care Unit (CAM-ICU) screening at admission and subsequent clinical confirmation according to DSM-5 diagnostic criteria (as detailed in the Section 2.4) [4,9,16]). Additional exclusion criteria were Glasgow Coma Scale (GCS) < 10 at admission; patients transferred to the IMCU after stabilization in the ICU (step-down care); and inability to provide informed consent.

### 2.3. Variables

At the time of IMCU enrollment, patient data were prospectively recorded in a structured electronic database. The following variables were recorded:

- Demographic data (age, sex, body mass index—BMI), and admission source (Emergency Department vs. step-up admission from a general ward).
- Medical history characteristics: Medical history variables were collected at IMCU admission, including cardiovascular, respiratory, renal, hepatic, metabolic, neurological, psychiatric, and oncological comorbidities. Comorbidity burden was assessed using the Charlson Comorbidity Index (CCI), analyzed both as a continuous variable and according to predefined thresholds (>2 points and >6 points). Additional variables included active oncological treatment and end-stage disease.
- Pre-admission clinical status: comorbidity burden was quantified using the CCI. Functional impairment was defined as any degree of dependence in activities of daily living, operationalized as a Barthel Index < 100. Chronic cognitive impairment was defined as a previously documented diagnosis of dementia or chronic cognitive decline reported in the patient’s available medical documentation and recorded at IMCU admission by trained nursing staff. Alcohol use disorder (AUD) was defined as a previously documented clinical diagnosis consistent with DSM-5 criteria and recorded at IMCU admission based on review of the available medical documentation. The Barthel Index components included feeding, bathing, personal hygiene, dressing, bowel and bladder continence, toilet use, transfers (bed-to-chair), mobility on level surfaces, and stair climbing. Reduced mobility was defined as the need for assistance with ambulation, including assistance from another person, use of walking aids (e.g., walker or cane), or inability to ambulate independently, either indoors or outdoors. End-stage disease was defined as advanced malignancy with an estimated life expectancy of less than three months or terminal organ failure. Limitation of intensive treatment was defined as documented restriction of escalation to intensive care, based either on physician judgment or on the patient’s documented preferences regarding treatment intensity.
- Clinical severity at IMCU admission: assessed through the National Early Warning Score (NEWS), Sequential Organ Failure Assessment (SOFA), and APACHE II scores.
- Number of organ failures at admission: total number of organs in a state of failure at admission.
- Need for advanced respiratory support: requirement for non-invasive ventilation (NIV) or high-flow nasal cannula (HFNC) oxygen therapy at admission.
- Use of vasoactive agents: administration of norepinephrine, epinephrine, dobutamine, or dopamine for persistent hypotension unresponsive to initial fluid resuscitation.
- Limitations of intensive treatment: physician-reported presence of conditions associated with exclusion from escalation to intensive care.

### 2.4. Outcomes

The primary study outcome was the development of an acute delirium episode within 96 h of IMCU admission. Delirium screening was systematically performed using the CAM-ICU at admission and subsequently twice daily during hospital stay, as well as in the presence of acute changes in mental status. Delirium screening and assessments were performed by nurses trained in the use of the CAM-ICU according to a standardized local protocol. A delirium episode was defined by the presence of at least one positive CAM-ICU assessment during the observation period, subsequently confirmed by a clinician according to DSM-5 diagnostic criteria, which include an acute alteration of attention and awareness associated with a global cognitive change from baseline, attributable to an underlying medical condition 4,9,16. Formal inter-rater reliability testing was not prospectively performed, and this has been acknowledged as a limitation.

The secondary outcome was 30-day mortality from IMCU admission. Vital status was assessed during hospitalization, at the time of discharge, and—if discharge occurred before day 30—through structured telephone interviews with the patient or a family member/caregiver.

### 2.5. Statistical Analysis

Continuous variables were described as the mean and standard deviation or as median and interquartile range, depending on the underlying distribution. Categorical variables were reported as percentages and counts. Univariate analyses were conducted using Fisher’s Exact test, Chi-square, Mann–Whitney U, and ANOVA tests, as appropriate.

To identify factors independently associated with the development of delirium, a multivariable logistic regression analysis was performed. The initial model included variables found to be associated with delirium in the preceding univariate analysis, avoiding the simultaneous inclusion of collinear variables representing the same pathophysiological domain. A backward stepwise selection procedure was used to obtain a parsimonious model and to reduce redundancy among clinically overlapping variables. Retention criterion was set at *p* < 0.05. Candidate variables were selected based on univariate associations and clinical plausibility, while avoiding simultaneous inclusion of highly collinear variables representing the same pathophysiological domain. Results were expressed as odds ratios (OR) with 95% confidence intervals (95% CI).

Classification and Regression Tree (CART) analysis was performed as an exploratory approach to identify hierarchical risk-stratification patterns and non-linear interactions among clinically relevant variables associated with the study outcomes. The model included the main variables identified as relevant from the multivariate model, with the aim of generating a clinically interpretable risk stratification. Decision trees were constructed using the Classification and Regression Tree (CRT) algorithm implemented in IBM SPSS Statistics, applying the Twoing impurity criterion for node splitting. Tree growth was restricted to a maximum depth of five levels, with a minimum parent node size of 50 observations and a minimum terminal node size of 20 observations. The minimum improvement required for node splitting was set at 0.001. Equal misclassification costs were applied, and prior probabilities were derived from the observed outcome distribution within the study cohort. Formal pruning procedures were not performed, as tree complexity was controlled a priori through conservative stopping rules. Similarly, no internal validation procedures were applied, since the objective of the analysis was hypothesis generation and exploration of clinically meaningful patterns rather than development and validation of a prediction model. This approach complemented the multivariable model by providing a hierarchical representation of variable interactions and identifying distinct patient subgroups with different risk phenotypes. The impact of delirium on 30-day mortality was subsequently assessed using a multivariable model adjusted for clinically relevant confounders identified in univariate analysis.

Interactions between risk factors and delirium were further explored using CART analysis, as described above.

All analyses were considered statistically significant at *p* < 0.05. Decision trees were constructed using IBM SPSS Statistics (version 31.0; IBM Corp, Armonk, NY, USA), while all other statistical analyses were performed using Stata (version 18; StataCorp LLC, College Station, TX, USA) and R (version 4.4.2; R Foundation for Statistical Computing, Vienna, Austria).

## 3. Results

**Figure 1:**
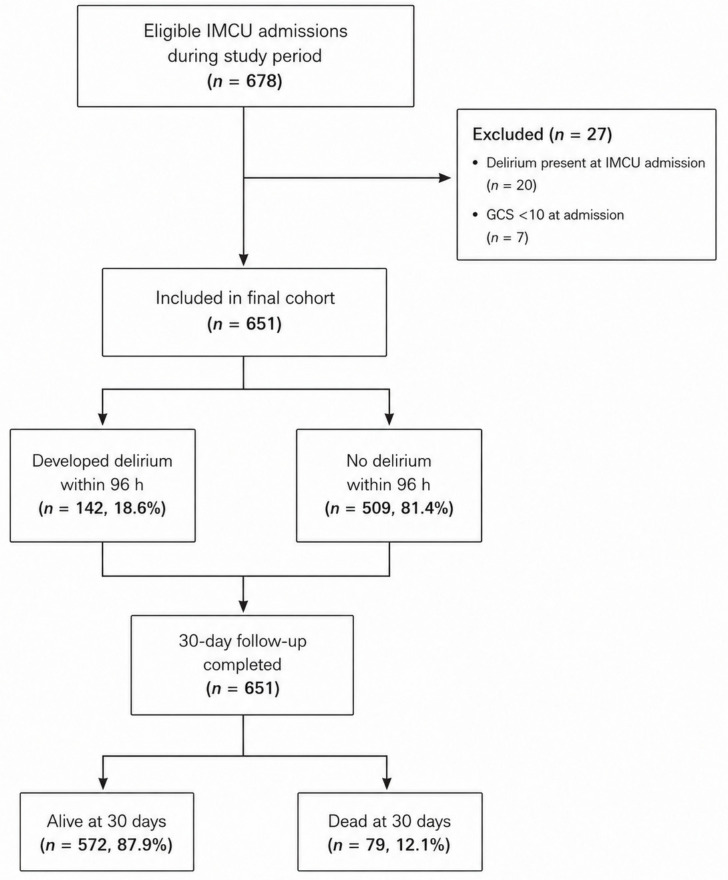
Flow diagram illustrating patient selection, exclusions, study cohort composition, delirium occurrence within the first 96 h after IMCU admission, and 30-day outcome assessment. Among 678 eligible IMCU admissions, 27 patients were excluded because of delirium already present at admission (*n* = 20) or severe impairment of consciousness (Glasgow Coma Scale < 10; *n* = 7), resulting in a final cohort of 651 patients. Delirium developed in 142 patients (18.6%) within the first 96 h after admission. Complete 30-day follow-up was available for all included patients.

A total of 651 patients admitted to the IMCU for an acute condition not previously stabilized in the ICU were included (7 patients were excluded due to GCS < 10 at admission and 20 due to delirium already present at admission). During the first 96 h of IMCU stay, 18.6% of patients (142/651) developed delirium. The study flow and patient selection process are summarized in Figure 1. Patient characteristics are summarized in Table 1.

Patients who developed delirium were older and male sex was more represented. Among medical history characteristics, alcohol use disorder, liver disease, and chronic obstructive pulmonary disease were significantly associated with the development of delirium, while the overall burden of comorbidities, expressed by the Charlson Comorbidity Index, showed no relevant differences between groups. Patients with delirium also showed greater baseline vulnerability, evidenced by a higher prevalence of institutionalization, more functional impairment, and chronic cognitive impairment. From the perspective of acute clinical severity, delirium was associated with higher values of the main severity scores, particularly NEWS, SOFA, and APACHE II. Moreover, patients with delirium more frequently showed signs of respiratory and metabolic compromise, such as PaO_2_/FiO_2_ ratio < 200 and hypercapnia. Finally, a greater number of organ failures and the need for non-invasive respiratory support (NIV) were associated with the development of delirium, while no significant differences emerged in relation to the presence of infection or other specific acute conditions (Table 1).

In the logistic regression model, several clinical factors assessed at IMCU admission were independently associated with delirium development. Age was confirmed as a significant determinant, with an additional 3.7% increase in risk per year (OR 1.037, 95% CI 1.012–1.064; *p* = 0.004), while male sex was associated with a higher risk compared to female sex (OR 1.749, 95% CI 1.080–2.833; *p* = 0.023). Among baseline conditions, alcohol use disorder emerged as one of the strongest independent risk factors (OR 3.592, 95% CI 1.660–7.771; *p* = 0.001), along with the presence of chronic cognitive impairment (OR 3.354, 95% CI 1.531–7.347; *p* = 0.002). COPD was also independently associated with the development of delirium (OR 1.682, 95% CI 1.002–2.824; *p* = 0.049). Regarding acute clinical severity, the NEWS score remained a significant risk factor, with a 17.7% increase in risk per point (OR 1.177, 95% CI 1.078–1.286; *p* < 0.001). In addition, the need for non-invasive ventilation was associated with a marked increase in delirium risk (OR 3.828, 95% CI 2.162–6.778; *p* < 0.001). Interestingly, the presence of acute respiratory distress at admission was inversely associated with delirium development in the adjusted model (OR 0.525, 95% CI 0.298–0.925; *p* = 0.026) (Table 2).

Decision tree analysis identified the need for non-invasive ventilation as the primary hierarchical determinant of risk, defining a subgroup of patients with the highest probability of developing delirium. Among patients not requiring ventilatory support, chronic cognitive impairment emerged as the main discriminating factor, reflecting increased baseline vulnerability.

**Figure 2:**
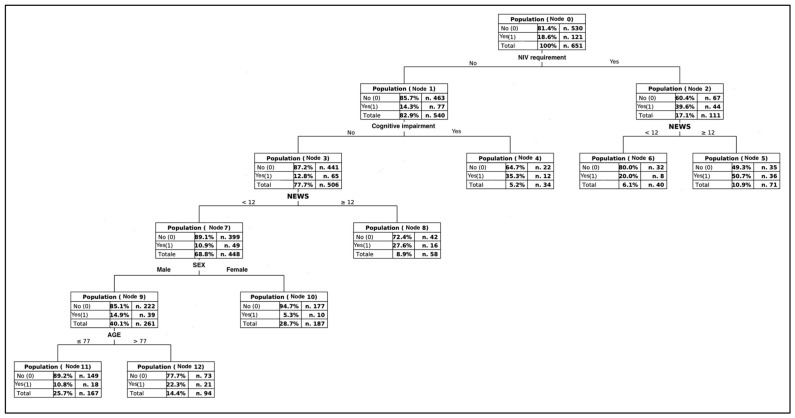
Decision Tree Analysis (CART) for delirium risk stratification in patients admitted to the IMCU. For each node, the distribution of the dependent variable is reported (0 = absence of delirium, 1 = presence of delirium). Tree depth = 5; total nodes = 13; terminal nodes = 7. Estimated resubstitution misclassification risk was 18.4%, overall correct classification rate was 81.6% (estimated risk 0.184 ± 0.015).

In the absence of cognitive impairment, delirium risk was further modulated by acute clinical severity, as measured by the NEWS score, with higher values associated with a greater likelihood of delirium. Overall, the decision tree revealed a hierarchical structure underlying delirium development, suggesting an interaction between acute physiological stress, pre-existing neurological vulnerability, and disease severity at IMCU admission (Figure 2).

Overall, 12.1% of patients (79/651) died within 30 days. Among patients who did not develop delirium, 30-day mortality was 9.8% (52/530), compared to 22.3% (27/121) among those who developed delirium (*p* = 0.001; univariate OR 2.640, 95% CI 1.578–4.418; *p* < 0.001).

Deceased patients showed older age, lower BMI, with a greater burden of comorbidities and worse acute conditions, with significantly higher values of CCI, NEWS, SOFA, and APACHE II. Mortality was also more frequent in patients admitted from general wards, with active cancer, ongoing chemotherapy, end-stage disease, institutionalization, reduced mobility, functional impairment, and chronic cognitive impairment. Among acute conditions at admission, acute kidney injury, ongoing infection, hypercapnia, lactate > 2, a higher number of organ failures, and the need for hemodynamic support were all associated with higher mortality (Table 3).

In the multivariable logistic regression model derived using backward stepwise selection, delirium remained independently associated with increased 30-day mortality (OR 2.530, 95% CI 1.349–4.747; *p* = 0.004).

Other independent risk factors included active malignancy (OR 3.814, 95% CI 1.945–7.480; *p* < 0.001), limitation of intensive care treatment (OR 4.608, 95% CI 2.545–8.345; *p* < 0.001), and the presence of three or more organ failures (OR 2.320, 95% CI 1.317–4.088; *p* = 0.004).

Indicators of respiratory and metabolic impairment, such as hypercapnia (OR 2.224, 95% CI 1.129–4.380; *p* = 0.021) and elevated lactate levels (OR 1.952, 95% CI 1.069–3.564; *p* = 0.029), also contributed to risk stratification. Age was independently associated with mortality (OR 1.038 per year; 95% CI 1.007–1.071; *p* = 0.017), whereas admission source was inversely associated with the outcome (OR 0.299, 95% CI 0.157–0.568; *p* < 0.001) (Table 4).

**Figure 3:**
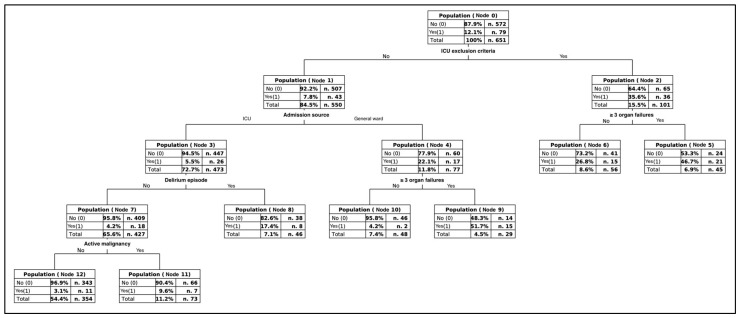
Decision Tree Analysis (CART) for 30-day mortality risk stratification in patients admitted to the IMCU. For each node, the distribution of the dependent variable is reported (0 = survivors, 1 = deceased). Tree depth = 4; total nodes = 13; terminal nodes = 7. Estimated resubstitution misclassification risk was 12.0%, overall correct classification rate was 88.0% (estimated risk 0.120 ± 0.013).

The decision tree analysis revealed a hierarchical structure in the determination of 30-day mortality, in which the main initial determinants were represented by conditions of severity and therapeutic limitations, particularly the presence of exclusion criteria from intensive care. Within these high-risk subgroups, additional factors—including general ward admission source, active malignancy, and number of organ failures—further refined prognostic stratification. In this framework, delirium emerged as a discriminative factor at deeper levels of the tree, contributing to the identification of subgroups with different mortality risks even at comparable levels of clinical severity. This pattern suggests that delirium integrates into the continuum of clinical complexity not merely as a marker of severe illness, but as a factor capable of further modulating risk within already vulnerable patient categories (Figure 3).

## 4. Discussion

In this prospective study conducted in an Intermediate Medical Care Unit (IMCU), we observed that approximately one in five patients develops delirium within the first 96 h after admission, confirming that this setting represents a high-risk environment, intermediate between general wards and intensive care units. The prevalence of delirium in IMCUs appears to be intermediate between that historically described in general wards (approximately 10–15%) [2,17] and in ICUs (40–50%) [18], However, recent evidence suggests that, due to increasing median age and comorbidity burden, the prevalence of delirium in general wards may now be closer to that observed in our study (approximately 20%) [19].

The IMCU setting represents a relatively novel context in delirium research, as it combines epidemiological risk characteristics typical of general wards (e.g., advanced age, comorbidities, high care needs) with negative environmental factors (e.g., continuous monitoring, noninvasive ventilation) more commonly seen in higher-intensity care settings. To date, structured policies for active delirium screening and prevention are not routinely implemented in IMCUs. The observation of prevalence rates comparable to general wards may be explained by the effectiveness of non-pharmacological preventive measures implemented by nursing staff (enabled by adequate staffing and nurse-to-patient ratios), or by the selection of patients, with exclusion of older, more comorbid, or functionally dependent individuals from escalation of care, as suggested by most recent literature [20,21].

Currently, no practical risk-stratification tools are available to guide clinicians in identifying, at the time of IMCU admission, patients at higher risk of developing delirium. In recent years, research efforts have mainly focused on diagnostic tools to accurately identify delirium once present; however, validated predictive tools applicable at enrollment in non-delirious patients are still lacking. This represents a significant gap in clinical practice. The present study was designed to provide an initial and exploratory response to this unmet clinical need.

The study findings support the conceptual model of delirium pathophysiology, which identifies the interaction between baseline vulnerability and acute illness severity as the core mechanism underlying delirium development [9]. Consistent with this model, the identified factors can be broadly categorized into two domains: predisposing and precipitating factors. Advanced age, functional dependency, low BMI, cognitive impairment, institutionalization were confirmed as predisposing factors in the study population [4,22]. These elements are frequently considered components of frailty and collectively describe a profile of increased physiological and cognitive vulnerability [22], highlighting the importance of a shared, multidisciplinary vulnerability assessment jointly conducted by physicians and nurses at IMCU admission to identify patients at higher risk of delirium during hospitalization.

Among predisposing factors, alcohol use disorder (AUD) and pre-existing cognitive decline had the strongest impact on delirium risk, consistent with findings across different settings and populations [23]. The role of chronic obstructive pulmonary disease (COPD) is also noteworthy, potentially reflecting a dual mechanism: increased baseline vulnerability and a higher propensity to develop blood gas abnormalities (particularly hypercapnia), which are known to promote acute cognitive dysfunction.

The association between delirium and respiratory distress or the need for respiratory support has not been consistently demonstrated in non-intubated patients. In our study, the need for non-invasive ventilation (NIV) emerged as one of the strongest determinants of delirium risk, both in multivariable analysis and in the decision tree model. This finding is clinically relevant and likely multifactorial: greater disease severity or hypoxemia as a central pathophysiological process, patient–ventilator asynchrony and discomfort, the need for sedative medications associated with delirium, as well as sleep disturbances and sensory deprivation related to the device. The CART analysis reinforces this finding, showing NIV as the primary decision node in risk stratification. This suggests that, in the IMCU setting, patients with respiratory failure requiring NIV should be considered a priority target for preventive strategies, including proactive screening and risk factor management. The inverse association between respiratory distress and delirium observed in the adjusted model should be interpreted cautiously. Because respiratory distress and NIV use are closely related clinical variables, simultaneous adjustment for both factors may have introduced statistical suppression effects or collider bias. Therefore, this finding should not be interpreted as evidence of a protective effect of respiratory distress, but rather as a potentially unstable association requiring confirmation in future studies specifically designed to address these interactions. An alternative and more speculative explanation relates to the definition of respiratory distress used in the present study, which was based primarily on an initial clinical assessment including inspection and vital signs. This approach may have grouped together patients with heterogeneous degrees of physiological impairment and different responses to treatment. It is conceivable that younger patients or those with greater physiological reserve may present with more overt clinical manifestations of respiratory distress that rapidly improve following appropriate organ support. Conversely, older and more vulnerable patients may exhibit less evident clinical signs despite severe underlying physiological derangement and a greater predisposition to delirium. From this perspective, clinically evident respiratory distress may not necessarily represent the most accurate marker of overall vulnerability to delirium when compared with more objective indicators of disease severity and organ dysfunction. However, this interpretation remains speculative and should be evaluated in future studies specifically designed to explore these relationships.

A particularly interesting and previously underexplored finding in this setting is the strong association between clinical severity scores (NEWS, SOFA, APACHE II) and delirium development. An observational study involving approximately 14,000 admissions demonstrated high specificity (99%) of the NEWS2 score in identifying delirium [24]. From a pathophysiological standpoint, these scores plausibly reflect systemic alterations such as cerebral hypoperfusion and hypoxia, both recognized contributors to delirium pathogenesis in critically ill patients [25,26]. The association with a rapid and simple score, routinely calculated in many clinical settings is particularly relevant, as it may improve recognition of delirium in resource-limited settings. Furthermore, the association between admission from a general ward as a step-up in care and delirium development underscores the link between clinical deterioration in acute patients and delirium pathophysiology, which is often underestimated [25,27]. Initial management of acute conditions in general wards without adequate intensive monitoring and treatment may lead to progressive accumulation of physiological stressors, contributing to an increased risk of delirium. The use of CART analysis allowed integration of logistic regression results, providing a hierarchical and clinically intuitive representation of delirium risk. Specifically, NIV use was identified as the primary discriminator, followed by cognitive impairment as a second-level modifier in patients not receiving NIV, and NEWS score as a final risk modulator. This approach highlights the non-linear interaction among risk factors and lays the groundwork for the development of simple, bedside-applicable risk stratification tools based on these variables.

Another key finding is that delirium was independently associated with increased 30-day mortality (OR ~ 2.5), even after adjustment for clinical severity and comorbidities. This is consistent with previous literature in comparable populations [5,28], but is particularly relevant in the IMCU setting, which remains under-investigated. Notably, in our multivariable mortality models, delirium emerged as an independent negative prognostic factor with a quantitatively greater impact than multiple organ failure (OR 2.32), hypercapnia (OR 2.22), or elevated lactate levels (OR 1.95). In other studies, the quantitative impact of delirium in critically ill patients has been comparable to that of persistent hypotension (SBP < 90 mmHg) or uncompensated hypoxemia (SpO_2_ < 90%) [29].

However, it remains unclear whether delirium is merely a marker of severe illness or a causal factor actively contributing to worse outcomes. Our data, together with CART analysis on mortality, suggest that delirium acts as a risk modulator in critically ill patients, further refining prognostic stratification within high-vulnerability subgroups. Potential mechanisms include impaired clinical management (poor compliance, device removal), increased complications (infections, falls), and systemic neuroinflammatory effects. Notably, in the decision tree, delirium appears as a finely discriminative factor, particularly in lower-risk patients or in those without stronger mortality-associated factors, reinforcing its prognostic significance. In this group of patients, the presence of delirium can lead to a meaningful increase in risk at the terminal node, acting as a quantitatively significant modifier. It is important to emphasize that our study included patients who were cognitively intact at admission, excluding those with delirium or altered GCS at baseline, unlike much of the existing literature [18], and included patients with heterogeneous acute conditions. This may partially explain the lower prevalence compared to ICU populations, where delirium is closely linked to acute stressors.

This study has relevant practical implications. Pharmacological treatments for delirium have been widely shown to have limited efficacy [30], primarily managing agitation or other care-related complications once delirium is manifest and fully developed, without improving short- and long-term outcomes [12,30]. Moreover, evidence-based non-pharmacological preventive strategies (e.g., A2F/ABCDEF bundles) [9,16] are resource and time-intensive and require substantial nursing effort. Early identification of high-risk patients may facilitate more efficient implementation of established non-pharmacological preventive measures. Whether specific high-risk populations may benefit from targeted pharmacological preventive strategies remains unknown and should be investigated in appropriately designed prospective studies [31,32]. To date, however, no pharmacological agent has demonstrated efficacy in delirium prevention, and current guidelines discourage their routine use in this setting [16]. It is also possible that previous negative trials may have been influenced, at least in part, by the inclusion of patients who had already developed delirium, rather than intervening within the appropriate preventive time window. The identification of readily available clinical variables in our analysis provides a preliminary framework for future validation studies aimed at developing IMCU-specific risk-stratification approaches [4,11]. Future research should determine whether these findings can be externally validated and incorporated into clinically applicable risk-stratification tools. If successfully validated, such tools could facilitate the early identification of high-risk patients and implementation of targeted preventive strategies, both non-pharmacological and pharmacological.

This study has several limitations.

First, although multivariable logistic regression and CART analysis allowed identification of admission-level factors associated with delirium and provided a clinically interpretable hierarchical representation of risk profiles, the study was not designed to develop or validate a formal prediction model. Measures of predictive performance, including discrimination, calibration, and model validation, were not primary objectives and were therefore not comprehensively assessed. Consequently, the identified associations and risk phenotypes should be interpreted as exploratory and hypothesis-generating. In addition, CART analyses were constructed using only variables previously selected through multivariable logistic regression. While this approach improved model parsimony and enhanced clinical interpretability, it may have limited the ability of the decision trees to identify additional predictors or alternative interactions among variables. Formal pruning procedures and internal validation techniques were not performed, which may have reduced the robustness and generalizability of the resulting trees. However, tree complexity was controlled a priori through conservative stopping rules, including restrictions on maximum depth and minimum node sizes. Importantly, CART was intentionally employed as an exploratory tool to provide a hierarchical representation of relationships among clinically relevant variables rather than to develop a clinically applicable decision-support system. Development of such tools would require additional methodological refinement, larger and more heterogeneous cohorts, and dedicated internal and external validation procedures. Therefore, the present findings should be considered a preliminary framework for future risk-stratification research in the IMCU setting rather than a validated predictive model ready for clinical implementation. Future studies should focus on formal model development and external validation across different IMCU settings before clinical implementation can be considered.

Second, its single-center design exposes it to potential biases related to local practices, limiting generalizability across different organizational settings. However, the use of standardized clinical assessments at admission and validated delirium screening tools may have mitigated these biases.

Third, delirium episodes were recorded as a binary outcome and were not further characterized according to clinical subtype (hypoactive, hyperactive, or mixed), duration, or severity. As these characteristics may carry important prognostic implications and influence clinical outcomes, future studies should incorporate a more detailed phenotypic characterization of delirium. Furthermore, potential modifiers such as sedative medications or pain control were not recorded, which may have influenced outcomes between baseline assessment and delirium onset. Nevertheless, such limitations are inherent to studies attempting to link static baseline variables with the development of a highly dynamic condition such as delirium. The aim was to provide clinicians with early indications to identify at-risk patients, not definitive predictions, but capable of raising awareness and promote clinical vigilance in high-risk patients.

Fourth, the CAM-ICU tool used for delirium screening was developed and validated in ICU settings rather than IMCUs but represented the most established tool available at the time of study design. In the absence of IMCU-specific widely validated tools, it was considered the most robust available option. Future studies should investigate the performance of newer IMCU-specific instruments such as CAM-IMC within this patient population.

Fifth, although delirium assessments were performed by trained staff using a standardized protocol and positive screenings were confirmed by a clinician, formal inter-rater reliability was not prospectively evaluated. Therefore, some degree of inter-observer variability cannot be excluded.

Sixth, variables collected at enrollment were predefined to describe disease severity in the IMCU population. Additional information on frailty, functional trajectories, and pre-admission clinical status could further enhance risk stratification. Seventh, in the present analysis, delirium was modeled as a binary variable occurring during the early IMCU stay, whereas mortality was assessed over a 30-day follow-up period. This approach does not fully account for the temporal relationship between delirium onset and death and may introduce temporal bias, including immortal time bias. Future studies should use time-dependent models or landmark analyses to better characterize the temporal relationship between incident delirium and subsequent mortality.

## 5. Conclusions

Delirium is a highly prevalent complication in the IMCU setting, resulting from the interaction between baseline vulnerability and acute illness severity, and is associated with a significant increase in 30-day mortality. The assessment of both vulnerability-related factors and those linked to acute disease and its management has the potential to identify, already at the time of IMCU admission, patients at risk of developing delirium, which may subsequently compromise overall clinical outcomes. Further studies are needed to better phenotype high-risk patients, to accurately identify those who may benefit from preventive strategies. To date, such interventions appear only partially effective, likely due to suboptimal selection of the target population in previous studies.

## Figures and Tables

**Table 1:** Univariate analysis of characteristics recorded at IMCU admission in patients stratified by subsequent development of an acute delirium episode. BMI, body mass index; CCI, Charlson Comorbidity Index; ICU, Intensive Care Unit; ABG, Arterial Blood Gas.

Variable	Total	No Delirium	Delirium	*p* Value
Patients, *n* (%)	651 (100)	530 (81.4)	121 (18.6)	
Age in years, median (IQR)	72.5 (12.5)	71.7 (13.1)	75.8 (9.6)	0.001
Sex, *n* (%)				
Male	370 (56.8)	290 (54.7)	80 (66.1)	0.025
Female	281 (43.2)	240 (45.3)	41 (33.9)	
BMI, median (IQR)	24.9 (22.1–28.6)	25.2 (22.2–29.4)	24.3 (21.6–26.3)	0.009
Admission source				
Ward	96 (14.7)	70 (13.2)	26 (21.5)	0.032
Emergency Department	555 (85.3)	460 (86.8)	95 (78.5)	
Medical history characteristics, *n* (%)				
Hypertension	432 (66.4)	355 (67)	77 (63.6)	0.532
Ischemic heart disease	119 (18.3)	96 (18.1)	23 (19)	0.796
Chronic Heart Failure (CHF)	181 (27.8)	146 (27.5)	35 (28.9)	0.822
Chronic vascular disease	126 (19.4)	103 (19.4)	23 (19)	1.000
Previous stroke/TIA	62 (9.5)	53 (10)	9 (7.4)	0.493
Alcohol use disorder (AUD)	47 (7.2)	29 (5.5)	18 (14.9)	0.001
Diabetes	185 (28.4)	155 (29.2)	30 (24.8)	0.372
Liver disease	32 (4.9)	21 (4)	11 (9.1)	0.032
Chronic obstructive pulmonary disease (COPD)	149 (22.9)	105 (19.8)	44 (36.4)	<0.001
Chronic Kidney Disease (CKD)	139 (21.4)	118 (22.3)	21 (17.4)	0.269
Known psychiatric disorder	74 (11.4)	58 (10.9)	16 (13.2)	0.525
Active cancer	84 (12.9)	73 (13.8)	11 (9.1)	0.179
CCI, mean (SD)	5.3 (2.7)	5.3 (2.8)	5.5 (2.5)	0.399
CCI categories, *n* (%)				
>2	557 (85.6)	448 (84.5)	109 (90.1)	0.151
>6	287 (44.1)	233 (44)	54 (44.6)	0.919
Active oncological treatment, *n* (%)	50 (7.7)	48 (9.1)	2 (1.7)	0.004
End-stage disease, *n* (%)	97 (14.9)	80 (15.1)	17 (14)	0.886
Nursing home resident, *n* (%)	59 (9.1)	39 (7.4)	20 (16.5)	0.004
Reduced mobility, *n* (%)				
No	505 (77.6)	413 (77.9)	92 (76)	0.631
Yes	146 (22.4)	117 (22.1)	29 (24)	
Functional impairment, *n* (%)				
No	410 (63)	345 (65.1)	65 (53.7)	0.022
Yes	241 (37)	185 (34.9)	56 (46.3)	
Cognitive impairment, *n* (%)				
No	609 (93.5)	507 (95.7)	102 (84.3)	<0.001
Yes	42 (6.5)	23 (4.3)	19 (15.7)	
ICU exclusion criteria, *n* (%)	101 (15.5)	78 (14.7)	23 (19)	0.265
Acute event at admission				
Acute Kidney Injury (AKI)	224 (34.4)	187 (35.3)	37 (30.6)	0.323
Infection	394 (60.5)	318 (60)	76 (62.8)	0.607
Acute myocardial Infarction (AMI)	32 (4.9)	29 (5.5)	3 (2.5)	0.243
Acute respiratory distress, *n* (%)	264 (40.6)	202 (38.1)	62 (51.2)	0.010
NEWS at admission, mean (SD)	5.3 (3.1)	5.1 (3.1)	6.6 (2.9)	<0.001
SOFA score, mean (SD)	3.4 (1.9)	3.3 (1.9)	3.7 (1.9)	0.046
APACHE II score, mean (SD)	12.2 (4.7)	12.1 (4.8)	12.9 (4.2)	0.039
ABG parameters, *n* (%)				
PaO_2_/FiO_2_ ratio < 200	161 (24.7)	120 (22.6)	41 (33.9)	0.014
Acidosis	99 (15.2)	78 (14.7)	21 (17.4)	0.483
Hypercapnia	106 (16.3)	71 (13.4)	35 (28.9)	<0.001
Lactate > 2	176 (28.9)	136 (27.3)	40 (35.7)	0.084
Number of acutely failing organs, *n* (%)				
At least one organ	603 (92.6)	485 (91.5)	118 (97.5)	0.020
Two or more organs	390 (59.9)	312 (58.9)	78 (64.5)	0.304
Three or more organs	178 (27.3)	134 (25.3)	44 (36.4)	0.017
Need for active organ support, *n* (%)	550 (84.5)	439 (82.8)	111 (91.7)	0.012
Need for respiratory support with HFNC, *n* (%)	208 (32)	175 (33)	33 (27.3)	0.236
Need for respiratory support with NIV, *n* (%)	111 (17.1)	67 (12.6)	44 (36.4)	<0.001
Need for immediate hemodynamic support, *n* (%)	107 (16.4)	82 (15.5)	25 (20.7)	0.175

**Table 2:** Multivariable logistic regression analysis (backward stepwise) of factors found significant in the preceding univariate analysis for delirium risk in IMCU.

Variable	OR	95% CI	*p* Value
Age (per year)	1.037	1.012–1.064	0.004
Sex (male vs. female)	1.749	1.080–2.833	0.023
Respiratory distress at admission	0.525	0.298–0.925	0.026
NEWS (per point)	1.177	1.078–1.286	<0.001
Alcohol use disorder	3.592	1.660–7.771	0.001
Need for noninvasive ventilation (NIV)	3.828	2.162–6.778	<0.001
Chronic obstructive pulmonary disease (COPD)	1.682	1.002–2.824	0.049
Impaired cognitive status	3.354	1.531–7.347	0.002

**Table 3:** Univariate analysis of characteristics recorded at IMCU admission stratified by 30-day mortality. BMI, body mass index; CCI, Charlson Comorbidity Index; ICU, Intensive Care Unit.

Variable	Alive at 30 Days	Dead at 30 Days	*p* Value
Patients, *n* (%)	572 (87.9)	79 (12.1)	—
Age, median (IQR)	75 (16)	78 (13)	0.002
BMI, median (IQR)	25.4 (6.5)	22.8 (5.7)	<0.001
CCI, mean (SD)	5.18 (2.72)	6.66 (2.50)	<0.001
NEWS, mean (SD)	5.12 (3.13)	6.67 (2.69)	<0.001
SOFA, mean (SD)	3.24 (1.76)	4.32 (2.48)	<0.001
APACHE II, mean (SD)	11.87 (4.61)	14.56 (4.58)	<0.001
General ward admission source, *n* (%)	69/96 (71.9)	27/96 (28.1)	<0.001
Active cancer, *n* (%)	59/84 (70.2)	25/84 (29.8)	<0.001
Active chemotherapy, *n* (%)	36/50 (72.0)	14/50 (28.0)	0.001
End-stage disease, *n* (%)	72/97 (74.2)	25/97 (25.8)	<0.001
Nursing home resident, *n* (%)	43/59 (72.9)	16/59 (27.1)	0.001
Reduced mobility, *n* (%)	116/146 (79.5)	30/146 (20.5)	<0.001
Functional impairment, *n* (%)	196/241 (81.3)	45/241 (18.7)	<0.001
Impaired cognitive status, *n* (%)	29/42 (69.1)	13/42 (31.0)	0.001
ICU exclusion criteria, *n* (%)	65/101 (64.4)	36/101 (35.6)	<0.001
Acute Kidney Injury (AKI), *n* (%)	188/224 (83.9)	36/224 (16.1)	0.026
Ongoing infection, *n* (%)	336/394 (85.3)	58/394 (14.7)	0.012
Hypercapnia, *n* (%)	85/106 (80.2)	21/106 (19.8)	0.008
Lactate > 2, *n* (%)	140/176 (79.6)	36/176 (20.5)	<0.001
Two or more failing organs, *n* (%)	328/390 (84.1)	62/390 (15.9)	<0.001
Three or more failing organs, *n* (%)	136/178 (76.4)	42/178 (23.6)	<0.001
Need for noninvasive ventilation (NIV), *n* (%)	92/111 (82.9)	19/111 (17.1)	0.078
Immediate hemodynamic support, *n* (%)	85/107 (79.4)	22/107 (20.6)	0.004
Delirium, *n* (%)	94/121 (77.7)	27/121 (22.3)	<0.001

**Table 4:** Multivariable logistic regression analysis of factors found significant in the preceding univariate analysis for the risk of 30-day mortality. ICU, Intensive Care Unit. * Reference group in multivariable analysis: Emergency Department.

Variable	OR	95% CI	*p* Value
Delirium	2.530	1.349–4.747	0.004
ICU exclusion criteria	4.608	2.545–8.345	<0.001
Admission source *	0.299	0.157–0.568	<0.001
Active cancer	3.814	1.945–7.480	<0.001
Lactate > 2 mmol/L	1.952	1.069–3.564	0.029
Hypercapnia	2.224	1.129–4.380	0.021
Age	1.038	1.007–1.071	0.017
≥3 organ failures	2.320	1.317–4.088	0.004

## Data Availability

The data presented in this study are available on request from the corresponding author due to privacy/ethical restrictions.

## Abbreviations

ABG	Arterial Blood Gas
BMI	body mass index
CAM-ICU	Confusion Assessment Method for the Intensive Care Unit
CART	Classification and Regression Tree
CCI	Charlson Comorbidity Index
ED	Emergency Department
GCS	Glasgow Coma Scale
HFNC	high-flow nasal cannula
ICU	Intensive Care Unit
IMCU	Intermediate Medical Care Unit
NEWS	National Early Warning Score
NIV	non-invasive ventilation
SOFA	Sequential Organ Failure Assessment
